# Supplementary material for: A Hybrid Stainless-Steel SPME Microneedle Electrode Sensor for Dual Electrochemical and GC-MS Analysis
Source: Sensors (Basel). 2023 Feb 19;23(4):2317. doi: 10.3390/s23042317 (PMC9963686; doi:10.3390/s23042317)
Supplement: Supplementary file 1 [file sensors-23-02317-s001.zip › sensors-2184350-supplementary.pdf]

A Hybrid Stainless-Steel SPME Microneedle Electrode Sensor for Dual Electrochemical and GC-MS Analysis

Samuel M. Mugo *, Scott V. Robertson and Marika Wood

Department of Physical Sciences, MacEwan University, Edmonton, AB T5J4S2, Canada
* Correspondence: mugos@macewan.ca

Supporting Information

a) Silylated MN

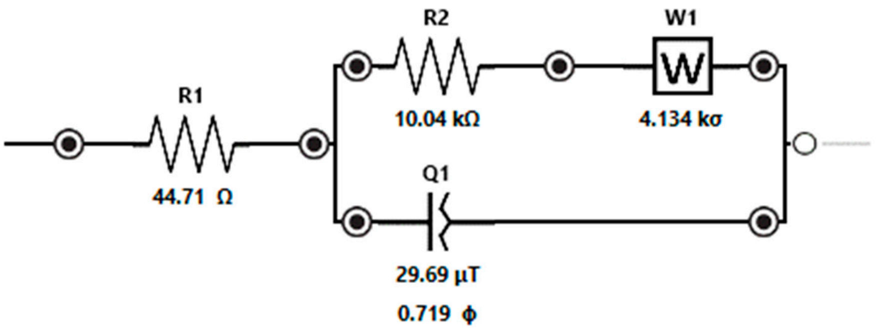

b) CNT/CNC coated MN

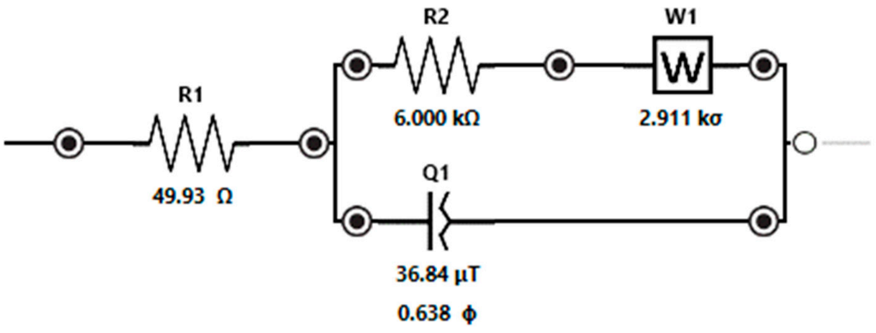

c) PANI@CNT/CNC SPME MN

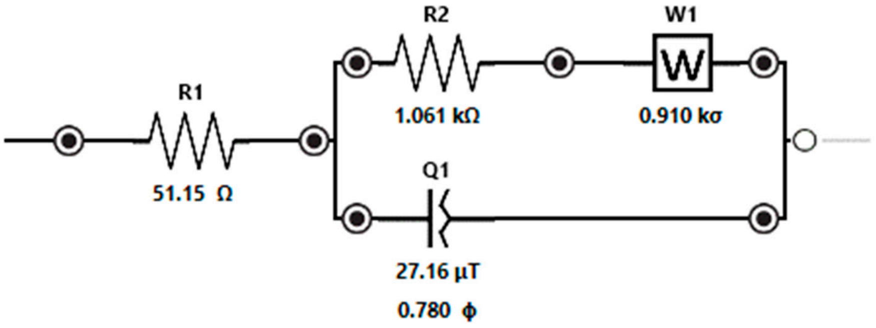

Figure S1. EIS circuit fitting for the different stages of fabrication for the PANI@CNT/CNC SPME MN.

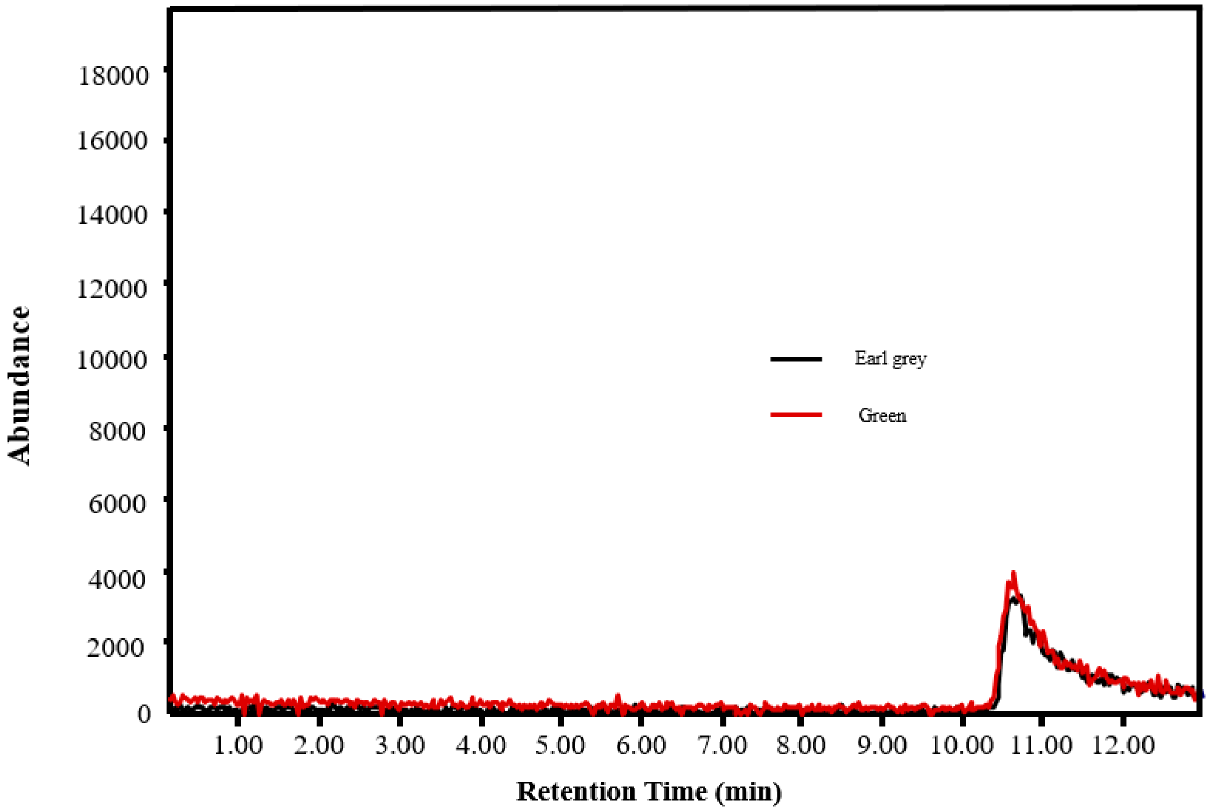

Figure S2. Overlaid extracted ion chromatograms (EIC) of GC-MS analysis of green and earl grey tea samples (m/z = 194 ± 0.30) acquired following SPME using the PANI@CNT/CNC SPME MN.
